# Effectiveness of waste-derived MIL type MOFs in removing PFOA and PFAS pollutants for environmental remediation

**DOI:** 10.1038/s41598-025-93854-0

**Published:** 2025-03-19

**Authors:** Mohamed A. Ismail, Anmar Ghanim Taki, Satish Kumar, Saad Sh. Sammen, Abdelfattah Amari, Arunkumar Bongale, Ozgur Kisi, Ali Salem

**Affiliations:** 1 Department of Chemical Engineering, College of Engineering, King Khalid University, 61411 Abha, Saudi Arabia; 2 Health and Medical Techniques College, Alnoor University, Mosul, Iraq; 3 Symbiosis Institute of Technology, Symbiosis International (Deemed University), Pune, India; 4 Department of Civil Engineering, College of Engineering, University of Diyala, Baqubah, Diyala Governorate 32001 Iraq; 5 Department of Civil Engineering, Lübeck University of Applied Science, 23562 Lübeck, Germany; 6 Department of Civil Engineering, School of Technology, Ilia State University, 0162 Tbilisi, Georgia; 7 School of Civil, Environmental and Architectural Engineering, Korea University, Seoul, 02841 South Korea; 8 Civil Engineering Department, Faculty of Engineering, Minia University, Minia, 61111 Egypt; 9 Structural Diagnostics and Analysis Research Group, Faculty of Engineering and Information Technology, University of Pécs, Pécs, Hungary

**Keywords:** Metal organic framework, PFOA removal, Water treatment, Waste materials, Environmental sciences, Environmental chemistry, Environmental impact

## Abstract

Elimination of perfluorooctanoic acid (PFOA), a persistent pollutant that is toxic to human and ecosystem health, is important. In this study, three adsorbents, C-101, W-101, and NW-101, were evaluated. W-101 was modified by diamine ethyl modification to enhance the number of PFOA adsorption sites. The results showed that W-101 (42.7 mg g^−1^) had better PFOA adsorption capacity than C-101 (12.3 mg g^−1^), and NW-101 (698.4 mg g^−1^) was the best. The Langmuir model correctly described the isotherms of PFOA adsorption, and the pseudo-second-order kinetic model fitted the process. NW-101 exhibited an excellent adsorption efficiency, as it reached the equilibrium within 7 min, and also revealed higher reusability due to the stable structure of the amine-grafted structure; therefore, NW-101 proved very efficient in PFOA removal. The new method used the bark of poplar trees to prepare MIL-101(Cr) adsorbents with surface areas of 3341, 2767, and 2374 m^2^ g^−1^ for C-101, W-101, and NW-101, respectively. This cost-effective, eco-friendly method utilizes renewable raw materials, minimizes environmental impact, and represents a significant advance in PFOA removal and thermal material research.

## Introduction

Recently, the issue of environmental pollution has received increased attention from researchers worldwide^1–3^. Specifically, researchers have been working hard to identify and counter those pollutants that have caused serious long-term risks to the environment and human health^4–6^. Among various environmental pollutants, PFAS has gradually become a major concern due to its abnormal persistence in biological systems and hazardous effects on ecological functions and human health^7^. PFASs are a series of man-made compounds characterized by fully or partially fluorinated carbon backbones and are extremely difficult to degrade by chemical and thermal means^8^. It is widely recognized that the C–F bond dissociation energies of PFASs are significantly higher than those of C–H bonds, making PFASs ultra-strongly resistant to hydrolysis and biodegradation^9^. PFASs usually contain hydrophobic fluorocarbon chains and various hydrophilic functional groups, allowing them to retain strong bioavailability and bioaccumulation potential in hydrophobic or hydrophilic environments^9^.

PFASs have been extensively utilized over the past 70 years in industrial applications, including agriculture, medicine, food packaging, non-stick coatings, fire-fighting foams, and lubricants, leading to their uncontrolled distribution and persistence in the environment. They have been used only considering economic purposes without exception, without the obligation for global monitoring. They pose a serious threat to human beings’ health and long-term ecological environment^10^. Since the 1960s, PFASs have been internationally reported to exist in surface water species, drinking waters, soil and biota worldwide, which embodies the problem of PFASs in the environment being strictly and effectively controlled^10–12^. Among these compounds, perfluorooctanoic acid (PFOA) is of particular concern to environmental experts because of its recalcitrant nature, as it is extremely persistent and problematic for humans due to its adverse effects on hematological, lymphatic, renal, and causing carcinogenic health problems^13^.

In this context, developing economically viable and advanced technologies for eliminating PFOA from aqueous solutions is highly necessary^14^. Although several techniques have been discussed in the literature to eradicate PFOA pollution from water bodies successfully, only a few of them have been reported to be cost-effective and advanced. The notable examples of PFOA treatment methods adopted from the scientific literature include electrochemical treatments, photocatalytic degradation, chemical reduction, adsorption, ultrasonication, biotreatment, and chemical oxidation^15–21^. Among all these techniques, adsorption is one of the most promising and widely accessible technologies because it is easy to implement, cost-efficient, and particularly effective in removing PFOA from water given that high-capacity adsorbents are used^22–24^.

Traditional adsorbents suffer from shortcomings like cost, selectivity, efficacy, and kinetics, calling for new approaches^25,26^. Among the progressive methodologies, metal–organic frameworks (MOFs) are currently the most attractive; 3D crystalline porous materials have excellent PFOA uptake capabilities^27–29^. MOFs form strong coordination bonds between metal clusters and multi-topic organic linkers, leading to highly customizable frameworks^30–32^. Among several MOFs, MIL-101(Cr) is recognized for its properties as host material: high porosity, thermal stability, and chemical resistance^33^. Up to the present, the conventional synthesis of MIL-101(Cr) has been conducted through solvothermal routes by utilizing terephthalic acid and high-purity Cr(III) in the presence of hydrofluoric acid (HF)^34^. HF is an obligatory constituent that not only helps in crystal growth and crystallinity improvement but also plays a crucial role in MOF formation; it could, however, be a serious health and environmental hazard and is inappropriate for large-scale production^35^.

Addressing the global challenge of waste management requires innovative solutions to combat the growing plastic pollution problem^36^. The socio-economical synthesis of MIL-101(Cr) from plastic waste has gained significant attention, as it opens a pathway for addressing plastic pollution in our biosphere^37^. In this respect, using cheap organic linkers comprised of recycled plastic waste (e.g., polyethylene terephthalate-based (PET)) appears promising, as PET waste accounts for a major portion of the global plastic waste^38,39^. Terephthalic acid (BDC; C_8_H_6_O_4_) can be derived from waste PET, vital in synthesizing MOF-based materials, and is an attractive way to curtail waste PET^40^. Additionally, high-quality MOF MIL-101(Cr) is typically obtained in the laboratory using pure Cr(III) source, as this guarantees keeping track of and controlling the metal-to-linkers ratio during the synthesis of the MOFs^41^. Nevertheless, the high cost of these chemicals makes the large-scale production of MOFs unfeasible. Therefore, researchers are looking for low-grade and readily available Cr sources to be used as the metal precursor in synthesizing Cr-based MOFs, as this continues to draw persuasive attention. To our knowledge, using Cr(VI) contaminated industrial waste as the metal precursor in preparing Cr-based MOF has not been reported. If this is realized, it may offer a one-pot means for the synthesis, providing a bottom-up approach to designing cost-effective Cr-based MOFs, and also, if the Cr metal source is of the large-scale industrial waste, it would offer an additional environmental advantage by preventing the dispersal of Cr in the environment.

This research highlights a novel method for synthesizing MIL-101(Cr) using Cr(VI) wastewater and PET bottles as the raw materials for the metal and ligand source, respectively. This innovative approach addresses two significant environmental challenges: plastic pollution and chromium contamination. Nontoxic and hazardous dichromate is reduced by sodium sulfite, and the resulting Cr(III) solution is adopted as the metal source for MIL-101(Cr) synthesis without further purification. The pH of the synthesis solution is adjusted using HNO_3_ instead of the addition of the mineralizer, as in the conventional HF-induced method, remaining a lower synthesis pH. Low-purity Cr(III) can synthesize MIL-101(Cr) to avoid generating a high concentration of HF upon acidification for the preparation. The synthesized MIL-101(Cr) is systematically characterized and compared against conventional MOFs fabricated by high-purity Cr(III) sources, e.g., Cr(NO_3_)_3_, with the assistance of HF. Ethylendiamine is further grafted on MIL-101(Cr) to enhance the adsorption performance of the perfluorooctanoic acid (PFOA). The low-cost fabrication of MIL-101(Cr) using waste resources aiming at a waste-to-energy approach provides a much lower cost and environmental impact than conventional preparation; the new route is promising for preparing other MOF materials.

## Materials and methods

### Materials

The present study used the following reagents: nitric acid, 1,4-benzene dicarboxylic acid, *N*,*N*-dimethylformamide, chromium nitrate nonahydrate, ethanol and sodium sulfite. All chemicals were purchased from Merck and were of analytical grade with high purity. The PET water bottles were collected from local sources, and washed, and then size reduced to 2 mm × 2 mm before being used for further analysis. The various chemicals were used in their as received state without any further purification which had high purity and were analytical grade. 180 mL of wastewater sample containing Cr(VI) of the required concentration was collected from an electroplating process.

### Synthesis procedure of MIL-101(Cr) from waste sources and pure chemicals

The synthesis of MIL-101(Cr) was carried out in an acidic medium to reduce Cr(VI) to Cr(III) using Na_2_S_2_O_5_ at room temperature. The pH was first adjusted to 2.5 by adding HNO_3_. Then, 2.90 g Na_2_S_2_O_5_ was introduced into the mixture using a drop funnel over 1 h and kept stirred. The mixture containing Cr(III) can be used as a metal source to synthesize MIL-101(Cr).

The PET bottles were washed using DI water and dried for 6 h at 80 °C. The rigorous washing process was employed to eliminate any impurities, and the drying step was to make them perfectly dried and ready for subsequent usage. The dried bottles were cut into pieces having only 1 mm in size × 1 mm.

A weight of 4 g of PET was mixed with the Cr(III) solution obtained by the previous process in a 150 mL beaker. The mixture was stirred for 30 min and then transferred to a Teflon autoclave. After the Teflon autoclave was enclosed in a stainless steel vessel it was heated to 100 °C for 150 min, then 220 °C for 14 more hours. After the reaction was completed, the obtained solid material was filtered and washed with water, DMF, and ethanol at 75 °C. At the end, the product was oven-dried at 100 °C for 10 h. From now on, our obtained product is labelled as W-101. The synthesis of MIL-101(Cr) from pure chemicals used in C-101^33^, Cr(NO_3_)_3_ 0.9H_2_O and H2BDC in the presence of hydrofluoric acid without the scaffold of PET is named C-101^42^.

### Synthesis procedure of amine grafted MIL-101(Cr)

**Fig. 1:**
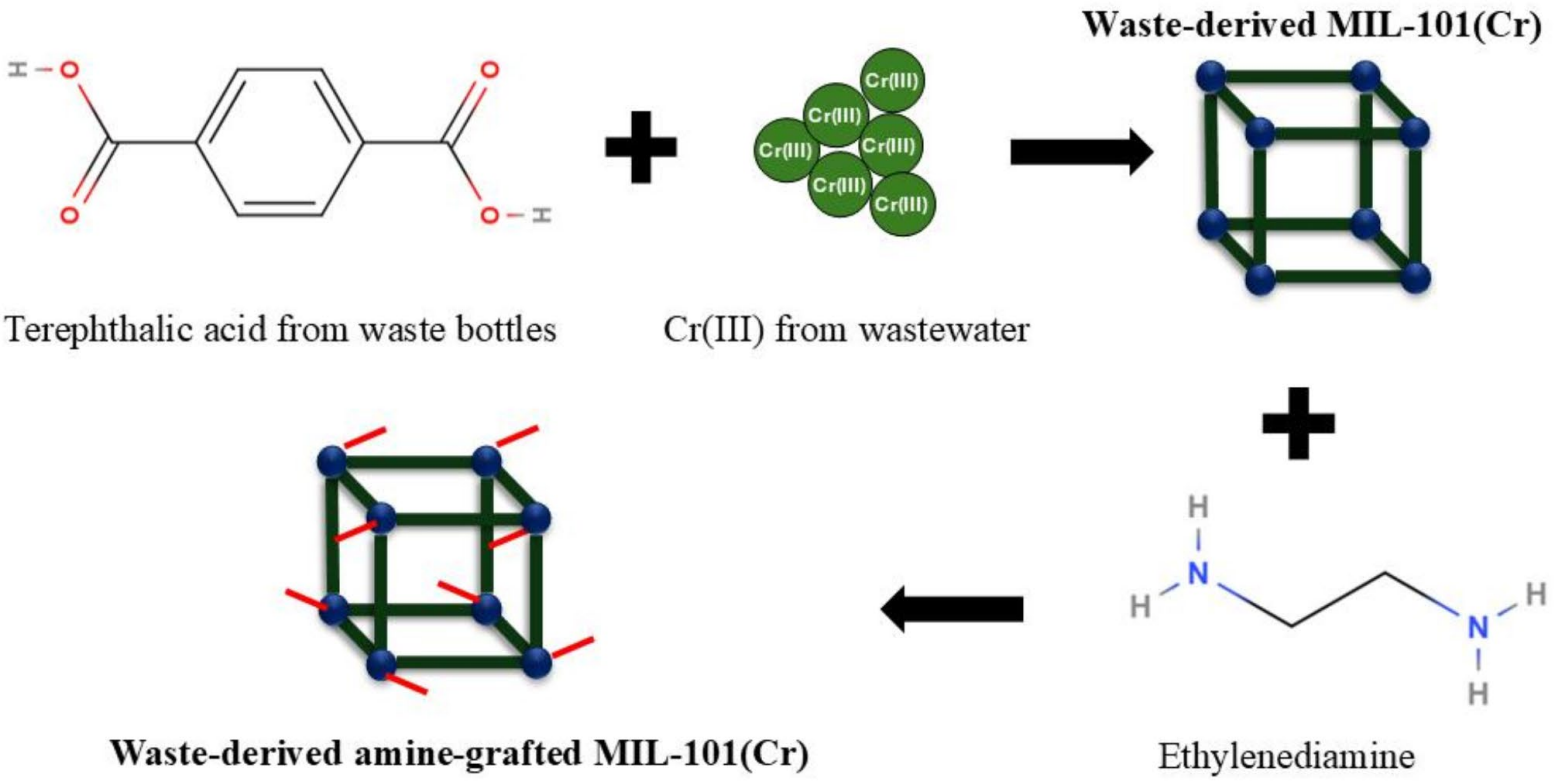
Schematic illustration of the synthesis procedure.

After the synthesis of W-101, the catalyst was activated in a high vacuum at 150 °C for 12 h. In the next step, 1 g of the obtained W-101 was added to 100 mL of hexane and sonicated in an ultrasonic bath for 30 min to obtain a fine suspension. Next, 0.25 mL of ethylenediamine was slowly introduced into the hexane suspension forming a jet from the end of the syringe, while the solution was vigorously stirred for 30 min. The resulting suspension was allowed to stand for 5 h while it was sealed, and then the remaining hexane was evaporated by opening the vial to the air. The green solid was dried at 160 °C in vacuo for 6 h. This procedure was performed to remove the solvent and any ED molecules adsorbed on the outer surface of W-101, finally leading to amine-grafted W-101, named NW-101 hereafter. The synthesis procedure is depicted schematically in Fig. 1.

### Materials characterization

The products, including W-101, NW-101 and C-101, were characterized via the Brunauer–Emmett–Teller method, X-ray crystallography, Fourier-transform infrared spectroscopy, Thermogravimetric analysis, and X-ray photoelectron spectroscopy.

### PFOA adsorption methodology

In batch experiments, 5 mg of W-101, NW-101, or C-101 was added to sample solutions (100 mL in volume and with PFOA content of 10 and 50 mg L^−1^) containing PFOA and then stirred with constant magnetic stirring at room temperature for about 1 day to reach equilibrium. The samples were further filtered to remove the solid adsorbent, determine the remaining PFOA content and evaluate the adsorption performance of each product. To investigate the influence of the initial pH (2, 4, 5, 6, 7, 8, 9), samples of PFOA with a concentration of 10 mg L^−1^ were prepared, and the solutions were adjusted to the desired pH value. To each of these solutions, 5 mg of the adsorbent was added. The samples were stirred to reach equilibrium, and the solid adsorbent was further filtered to determine the remaining PFOA content and confirm the effect of pH on the adsorption performance of each product. To investigate the impact of time, PFOA of different concentrations was added to the test solutions. The concentration of PFOA in the solutions was measured at different intervals. The steps were repeated for each adsorbent, and samples were taken. The adsorption study of the product was performed by using the following two equations:

1 $$Q_{e} \;\left( {\text{mg/g}} \right) = {{\left( {C_{i} - C_{e} } \right) \times v} \mathord{\left/ {\vphantom {{\left( {C_{i} - C_{e} } \right) \times v} m}} \right. \kern-0pt} m}$$

2 $${\text{PFOA removal percentage}} = {{\left( {C_{i} - C_{e} } \right) \times 100} \mathord{\left/ {\vphantom {{\left( {C_{i} - C_{e} } \right) \times 100} {C_{i} }}} \right. \kern-0pt} {C_{i} }}$$

Here, $C_{i}$ (mg L^−1^) is attributed to the initial PFOA content, while $C_{e}$ (mg L^−1^) ascribes the PFOA content after the elimination process. $v$ attributes the volume of the samples (L), and *m* represents the weight of adsorbent utilized for adsorption in g.

## Results and discussion

### Characterization of adsorbents

**Fig. 2:**
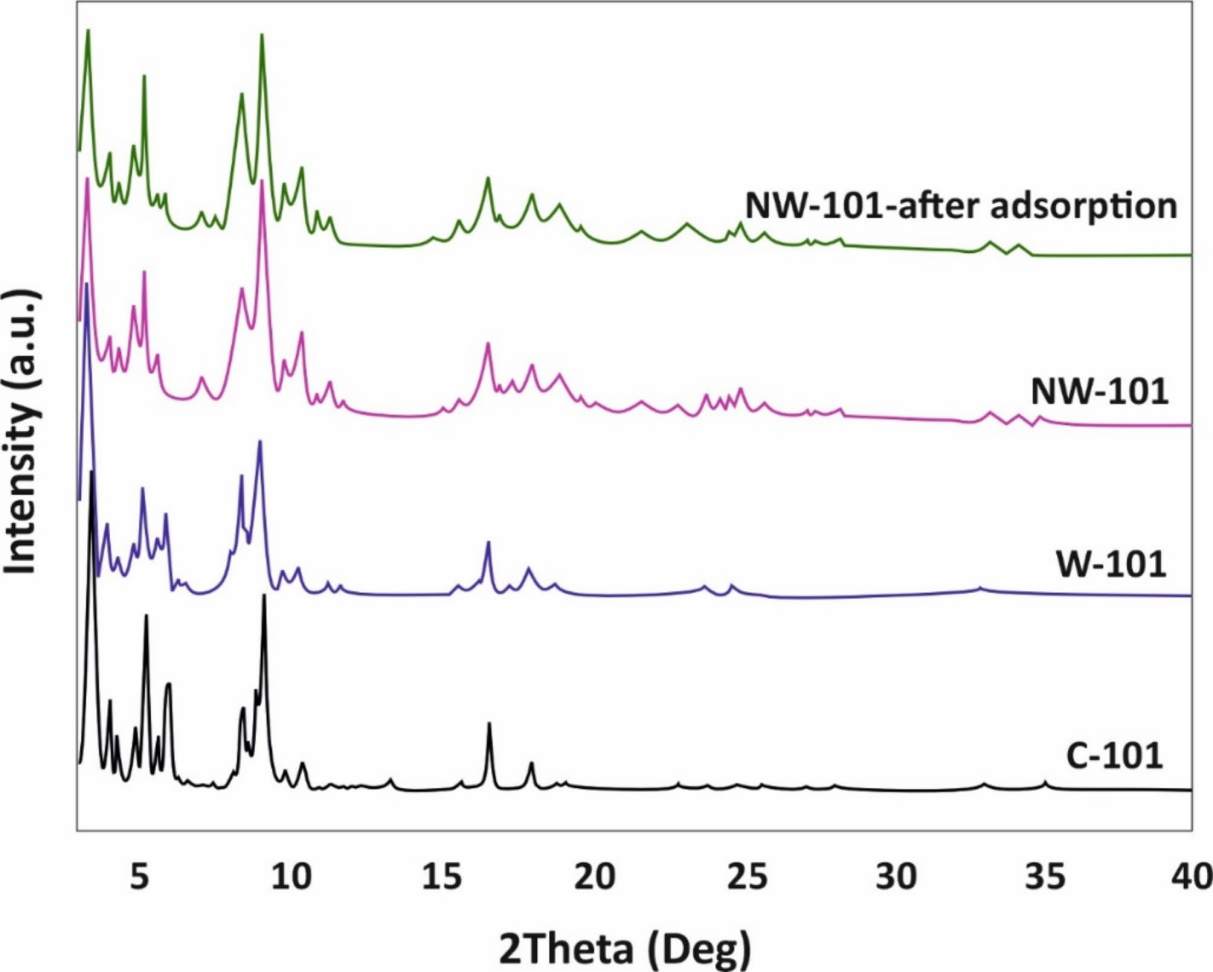
XRD pattern for C-101, W-101, and NW-101 after and before PFOA adsorption.

X-ray diffraction (XRD) patterns of MIL-101(Cr) adsorbents, i.e., C-101, W-101, NW-101 before and after adsorption, were used to discover their crystal structure and phase composition. As shown in Fig. 2, the W-101 and C-101 presented excellent peak fitting with seven peaks at 7.3°, 9.1°, 9.7°, 10.9°, 11.6°, 12.3°, 16.7° (Fig. 2). This confirms that the waste source chosen did not notably alter the crystal structure of the material, as it presented almost the same peaks as those reported by other authors^43^. Once again, the difference in the peak intensity displayed a small change in the phase composition. This is because the introduction of amine groups on the surface of the MIL-101(Cr) samples will cause the new XRD peaks to appear at 5.7°, 7.3°, and 10.4°, and the crystal structure and phase composition slightly change^43^. However, we measured by the XRD method that the XRD result of our sample was in good agreement with the above references and our measurement results. However, the phase composition of our sample was the same as that reported. As for the XRD patterns of the NW-101 after adsorption of PFOA, only the peak intensity at 9.1° and 11.6° decreased. Therefore, it could be considered that this method to absorb PFOA led to the adsorbent crystal structure changing a little. The crystalline framework of the adsorbent was maintained even after the adsorbent was put through the adsorption process, as indicated by the well-defined XRD peaks.

**Fig. 3:**
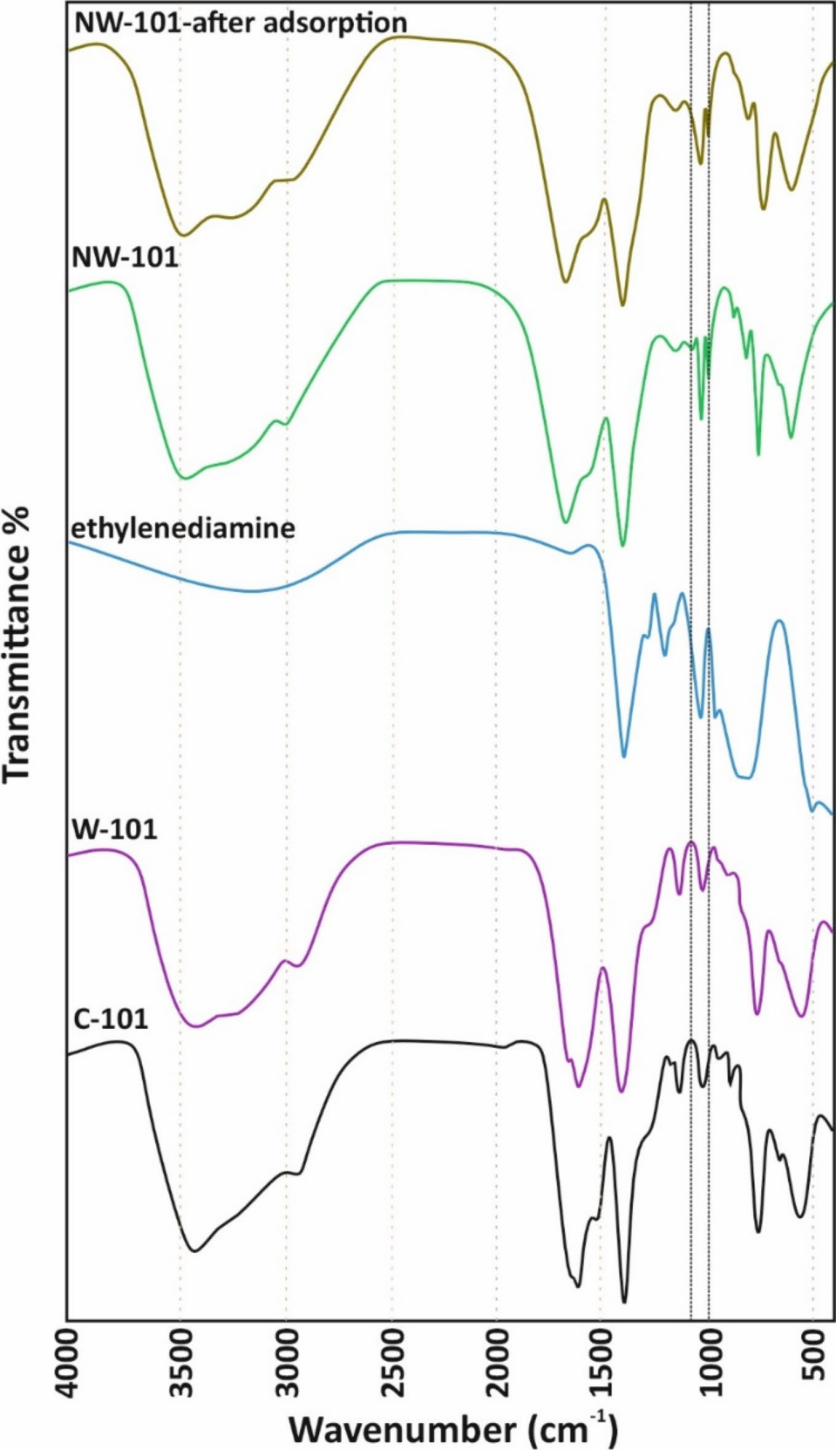
FTIR spectra for C-101, W-101, ethylenediamine, and NW-101 after and before PFOA adsorption.

Fourier transform infrared (FTIR) graph of the MIL101(Cr) adsorbents including C101, W101, NW101 before and after the adsorption study and the MIL101(Cr)-ED containing amine functional groups has been recorded and shown in Fig. 3. The FTIR graph of C101 and W101 exhibited the common peak patterns, which suggested that the introduction of agricultural waste-based precursors did not significantly disturb the functionalities of the substance. The dominating absorption bands in the FTIR graph of MIL101(Cr) fortitudes that C, W, and NW adsorbents around 3415, 1620, 1510, 1230 and 1060 cm^−1^ are attributed to the O–H and C=C stretching, C=C bending, C–O stretching, and C–H bending, respectively in the graph. These peaks match the MIL101(Cr) prepared from pure synthetic chemicals reported elsewhere^44^. An additional absorption band at around 1030 cm^−1^ in the FTIR spectrum of the MIL101(Cr)-ED suggested that the amine groups have been incorporated and confirmed the successful incorporation of the amine group^45^. It was observed that the absorption band of –NH_2_ in the FTIR graph of aminografted MIL101(Cr) samples was decreased in intensity following the PFOA adsorption process^45^. This reduction in the intensity of the –NH_2_ absorption band indicated that the amine groups were involved in retaining the PFOA molecules onto the sorbent surface. Moreover, the FTIR graph of the ethylenediamine (ED) revealed the dominating absorption band at 1030 cm^−1^, which confirmed the primary amine and C–N stretching. The presence of amine groups was also established by the absorption bands at 3300 and 1470 cm^−1^. The whole FTIR analysis supported the PFOA adsorption mechanism. The FTIR graph suggested that the amine groups were crucial in PFOA removal.

**Fig. 4:**
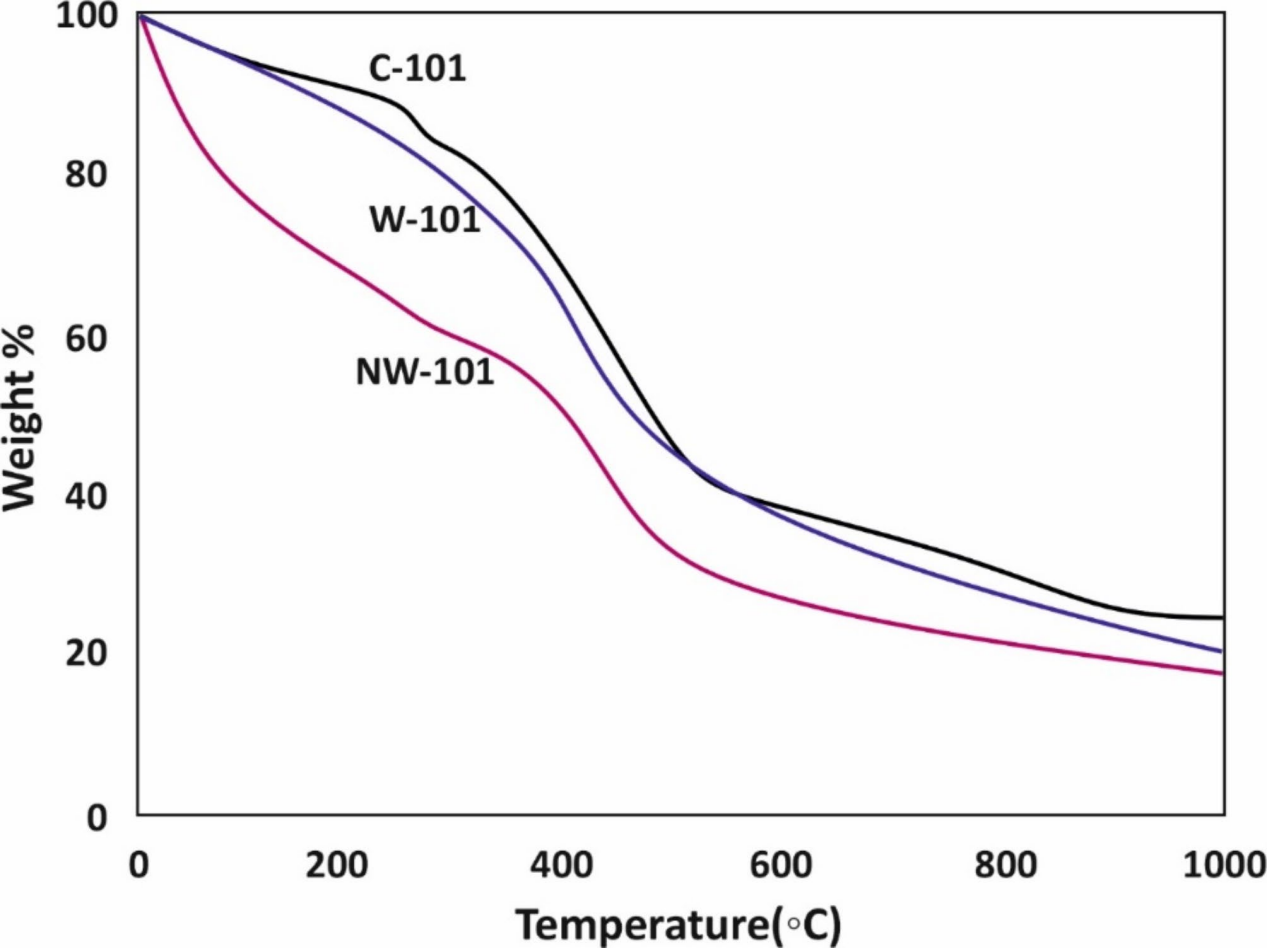
TGA analysis for C-101, W-101, and NW-101.

The heat-resistant properties of the MIL-101(Cr) adsorbents, C-101, W-101, and NW-101, were evaluated based on the thermogravimetric investigation (TGA). As shown in Fig. 4, the TGA profiles of W-101 and C-101 were almost similar in shape, which implies that the pyrolysis temperatures of W-101 were like that of the pure-chemical derived adsorbents. On the other hand, the TGA profile of NW-101 also shows a significant weight loss of approximately 20% before 100 °C owing to the removal of solvents present in the pore network. A continuous change in mass between 300 and 470 °C may be associated with the partial collapse of the framework and the removal of organic ligands. It shows that incorporating the grafting process only slightly affects the adsorbent’s heat resistance. At last, from the TGA analysis, we concluded that W-101 and NW-101, the synthesized MW-MIL-101(Cr), had similar heat-resistant performance to the pure-chemical adsorbents, which shows the prospect of applying the MW-MIL-101(Cr) derived from waste sources to the practical treatment of organic pollutants.

**Fig. 5:**
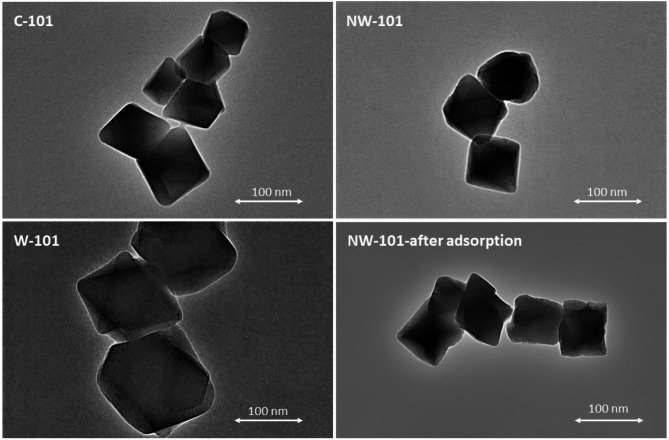
TEM images of C-101, W-101, and NW-101 after and before PFOA adsorption.

**Fig. 6:**
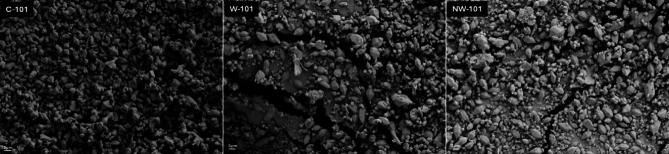
SEM images of C-101, W-101, and NW-101.

The TEM images in Fig. 5 show that the C-101 particles have an octahedral structure with sharp corners and edges, which is in good agreement with the reported works. The images also showed that the quality of W-101 is on par with that of C-101. The amine modification of the MOF does not affect the crystal size and structure of W-101. A little variation was noted in the crystalline structure of the MOF after the adsorption of PFOA, but the rigid skeleton it maintains is the Mo-Mo bond. SEM images also confirm the morphological properties of MOFs obtained from TEM imaging. Octahedral structure was observed for all MOFs with no significant changes after amination (Fig. 6).

**Fig. 7:**
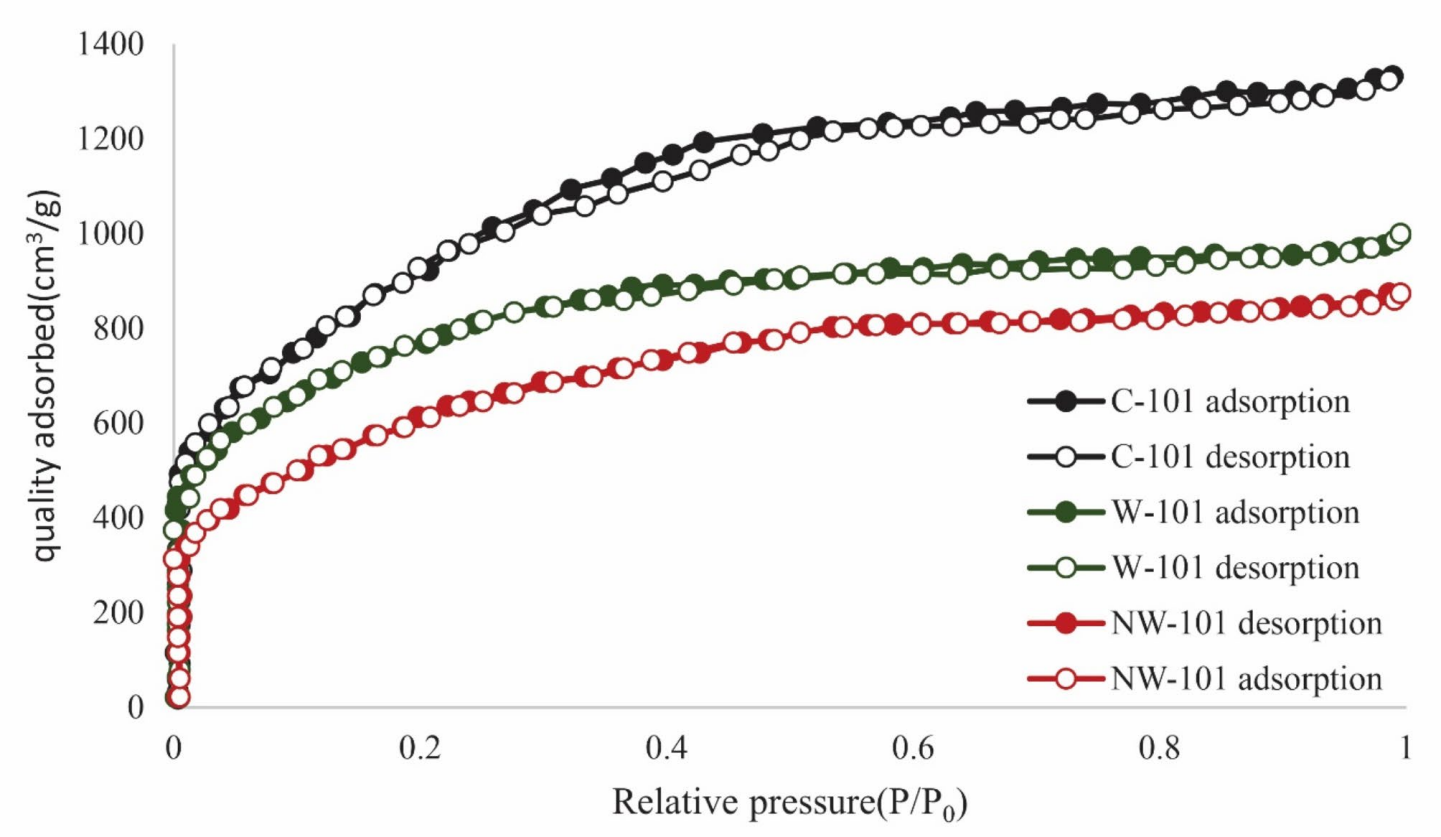
N_2_ adsorption–desorption for C-101, W-101, and NW-101.

**Fig. 8:**
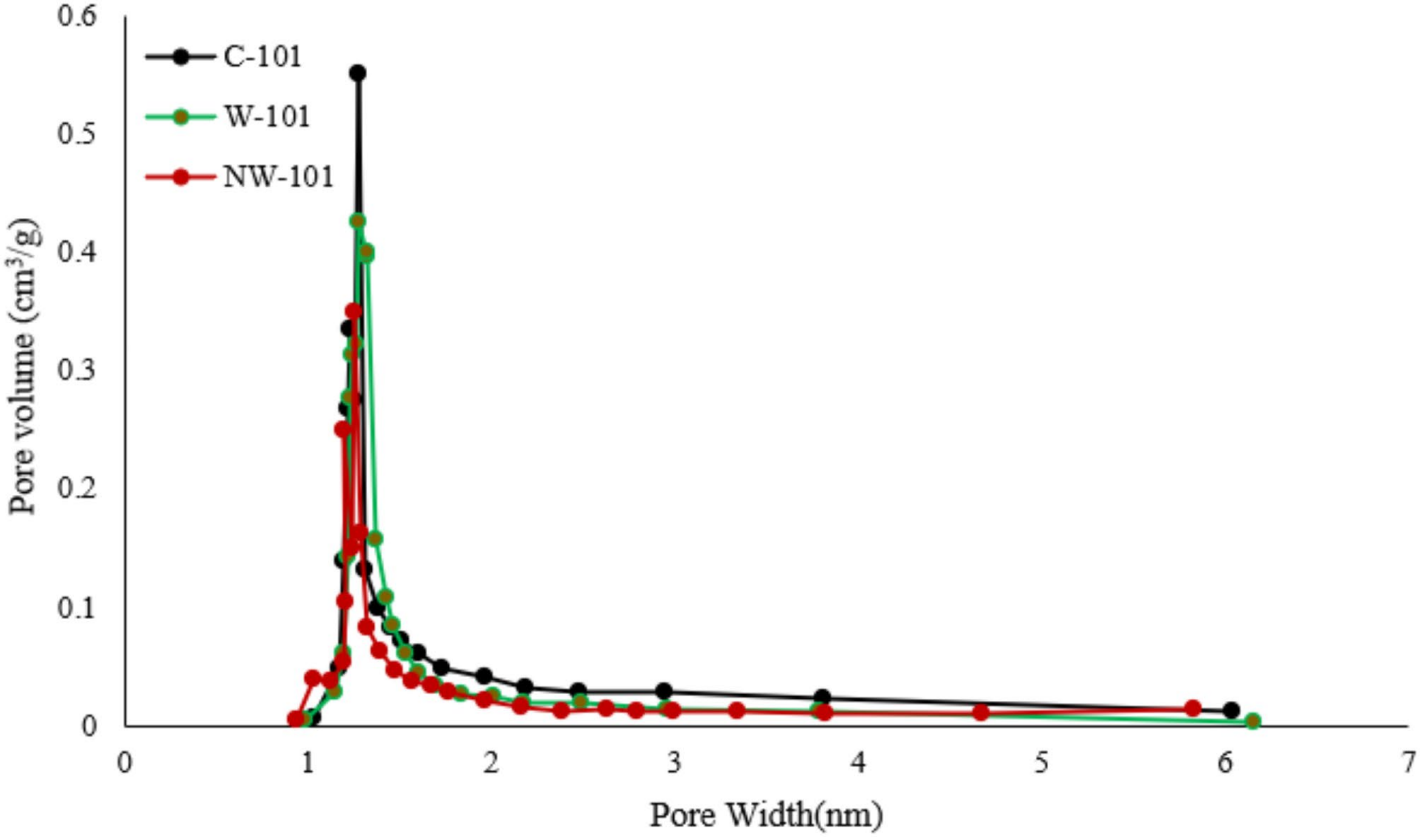
Pore size distribution diagram of C-101, W-101, and NW-101.

The adsorbents’ porosity and specific surface area were characterized using N_2_ adsorption–desorption isotherms at 77 K. As shown in Fig. 7, a remarkable type I isotherm was observed for all adsorbents, indicating their microporosity. It was acquired that all the samples gave a very high surface area and large pore volume, conforming to our anticipation. As known, the modification can cause the decrease of surface area and pore volume. It was confirmed from the data that both the surface area and pore volume of NW-101 doped notably from 3341 to 2374 m^2^ g^−1^ and from 1.43 to 1.18 cm^3^ g^−1^, respectively (Figs. 7 and 8). The pore size distribution and pore volume of the MOFs were determined based on non-local density functional theory (NLDFT) method from nitrogen adsorption–desorption isotherms performed at 77 K. The selection of the NLDFT model was done due to its ability to accurately reflect microporous structures of MOFs being central to this study. A remarkable decrease in pore volume was mainly due to the amine moieties the pores had previously filled. To further study the effect of amine grafting on pore size, the pore size distribution of adsorbents was investigated as shown in Fig. 7. The clear tendency was that the micropore volume of the modified adsorbent decreased by a large margin in contrast with the mesopores, which also indicated the remarkable decrease of specific surface area. These pointed to lessening pore size, meaning that the functional amine moieties had been successfully grafted onto the MIL-101 framework.

### Evaluation of PFOA removal: isotherms and kinetics

Conclusion to sum up, the present study was carried out to examine the efficiency of C-101, W-101, and NW-101 as adsorbents for the removal of PFOA from aqueous solution. Freundlich and Langmuir’s isotherms were used, and the pseudo-first and pseudo-second-order kinetic models were employed to fit the PFOA adsorption kinetics, which are provided in Eqs. (3), (4), (5) and (6).

Freundlich isotherm equation^46^:

3 $$Q_{e} = K_{f} C_{e}^{1/n}$$

Langmuir isotherm equation^47^:

4 $$Q_{e} = \frac{{C_{e} K_{l} Q_{m } }}{{1 + C_{e} K_{l} }}$$

Pseudo-first order kinetic equation:

5 $$Q_{t} = Q_{m} \left( {1 - e^{{ - K_{1} \times t}} } \right)$$

Pseudo-second order kinetic equation:

6 $$Q_{t} = \frac{{K_{2} \times Q_{m}^{2} \times {\text{t}}}}{{1 + K_{2} \times Q_{m} \times {\text{t}}}}$$

**Fig. 9:**
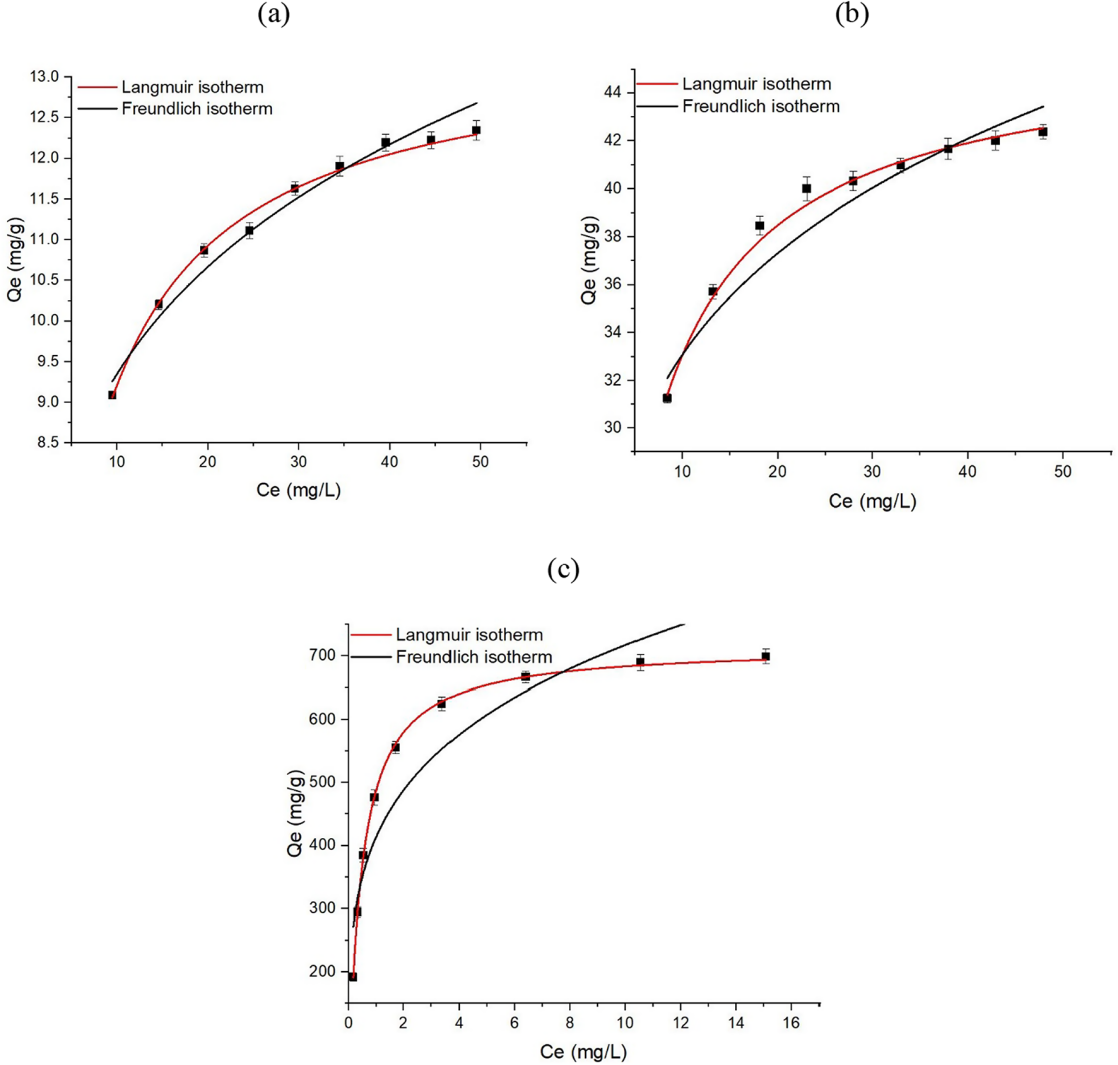
(**a**) Isotherm model for C-101, (**b**) isotherm model for W-101, and (**c**) isotherm model for NW-101.

**Fig. 10:**
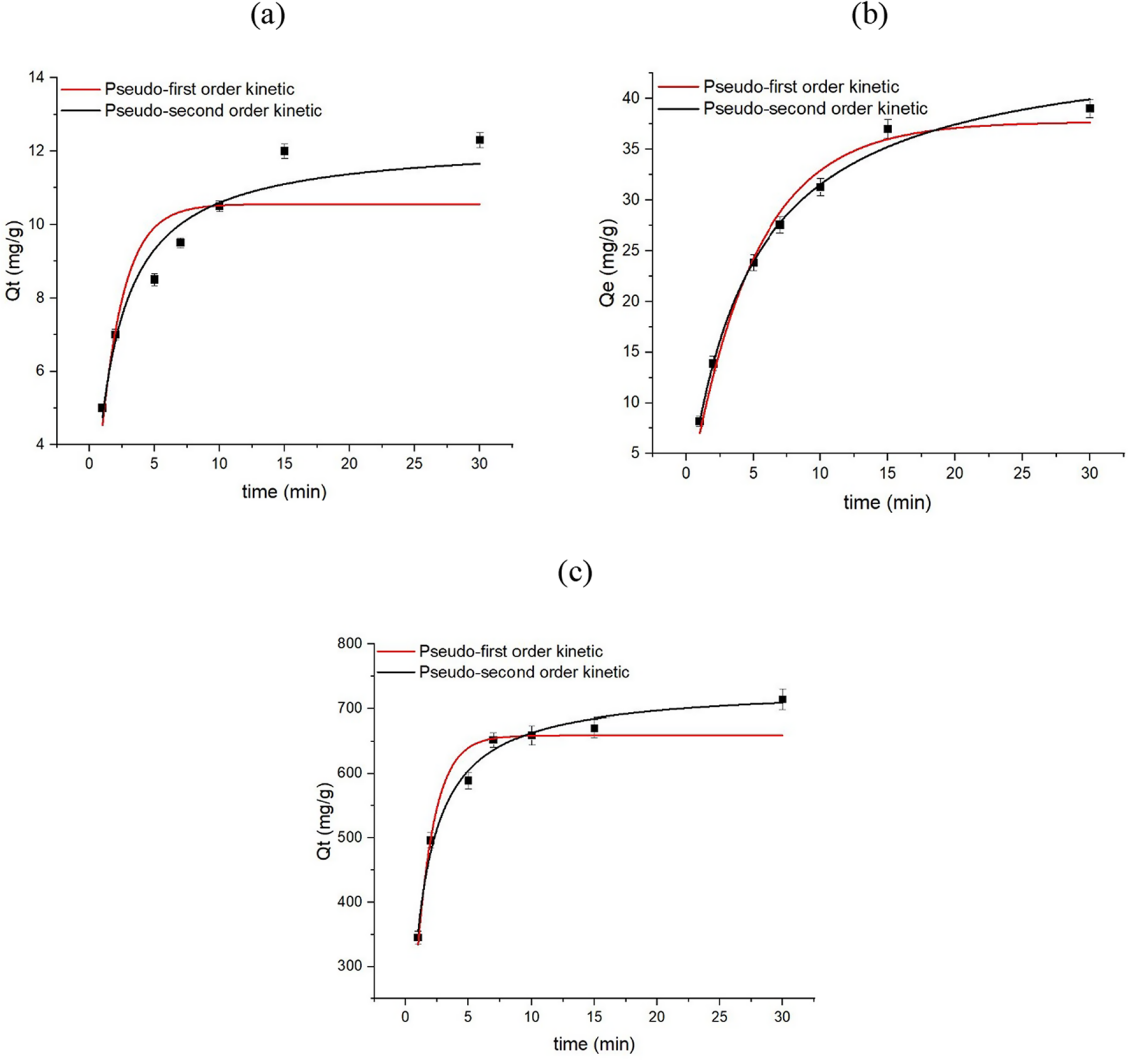
(**a**) Kinetic model for C-101, (**b**) kinetic model for W-101, and (**c**) kinetic model for NW-101.

The maximum removal capacities of PFOA on C-101, W-101 and NW-101 calculated using the Langmuir model were 13.6, 45.9 and 720 mg g^−1^, respectively (Fig. 9 and Table 1). However, actual removal amounts of PFOA were 12.3, 42.7 and 698.4 mg g^−1^, meaning the actual adsorption capacities on the materials were slightly lower than the theoretical capacities. Moreover, NW-101 showed the lowest PFOA adsorption capacity of all materials, and it attained equilibrium more rapidly. The pseudo-second order model was the most suitable for describing the PFOA adsorption process in all cases (Fig. 10 and Table 2). This indicates that chemisorption was the rate-determining step, according to^48^. With the Langmuir model, a better fit to the adsorption isotherm indicates that the adsorption process occurred on the homogenous surface of the adsorbent that the number of the adsorption sites is limited, and that the adsorption can only occur once on a particular site^49,50^. The results suggest that the MOF materials obtained in this study could be high-performance adsorbents for PFOA removal from wastewater. The PFOA removal capacity of the MOFs was compared to other MOFs from previous literature in Table 3. NW-101 was clearly the most efficient material among all MOFs, reaching higher PFOA removal capacity compared to the MOFs Kong et al.^51^ and Hu et al.^52^, which showed very low PFOA adsorption capacity of only 76.59 mg g^−1^ and 98.2 mg g^−1^, respectively.

**Table 1:** Isotherm limitations of C-101, W-101, and NW-101.

Adsorbents	Langmuir			Freundlich		
	*q*_*m*_ (mg g^−1^)	$K_{l}$ (L mg^−1^)	*R* ^2^	*n*	$K_{f}$ (mg g^−1^)	*R* ^2^
C-101	13.6	0.21	0.992	5.61	6.28	0.971
W-101	45.9	0.26	0.996	5.76	22.19	0.945
NW-101	720	2.13	0.999	4.77	432.91	0.871

**Table 2:** Kinetic limitations of C-101, W-101, and NW-101.

Adsorbents	Pseudo-first-order			Pseudo-second-order		
	*q*_*m*_ (mg g^−1^)	*K*_1_ (1/min)	*R* ^2^	*q*_*m*_ (mg g^−1^)	*K*_2_ (g mg^−1^ min^−1^)	*R* ^2^
C-101	10.56	0.56	0.855	12.27	0.051	0.966
W-101	37.7	0.21	0.986	46.16	0.0045	0.996
NW-101	659.15	0.7	0.954	734.1	0.0013	0.991

**Table 3:** Assessment of the PFOA removal capacities of currently documented MOFs.

Adsorbents	Q (mg g^−1^)	pH	Initial concentration	References
F-TiO_2_@MIL-125	76.59	–	1–50 μmol L^−1^	^51^
DUT-5-2	473.7	3	30 mg L^−1^	^52^
Zr-MOF/La-MOF	340/364	7	1–20 mg L^−1^	^53^
MIL-101(Cr)-PAM	492.7	5	0.1–0.6 mmol L^−1^	^33^
MIL-96-RHPAM2	340	–	1000 mg L^−1^	^54^
C-101	13.6	7	10–50 mg L^−1^	Present work
W-101	45.9	7	10–50 mg L^−1^	Present work
NW-101	720	7	10–50 mg L^−1^	Present work

### Evaluation of the pH influence on the PFOA elimination process: mechanism investigation

**Fig. 11:**
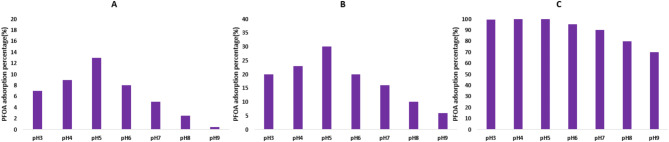
PFOA adsorption at a pH range of 3–9 for (**A**) C-101, (**B**) W-101, and (**C**) NW-101.

The role of the pH of the adsorption sample on the adsorption of PFOA onto MOFs is also one of the specific subjects in this investigation, as it is one of the critical parameters having a huge impact on the adsorption mechanism. Figure 11 illustrates that the pH was optimally controlled at 5 for the C-101 and W-101, and it was 3 for the NW-101, respectively.

**Fig. 12:**
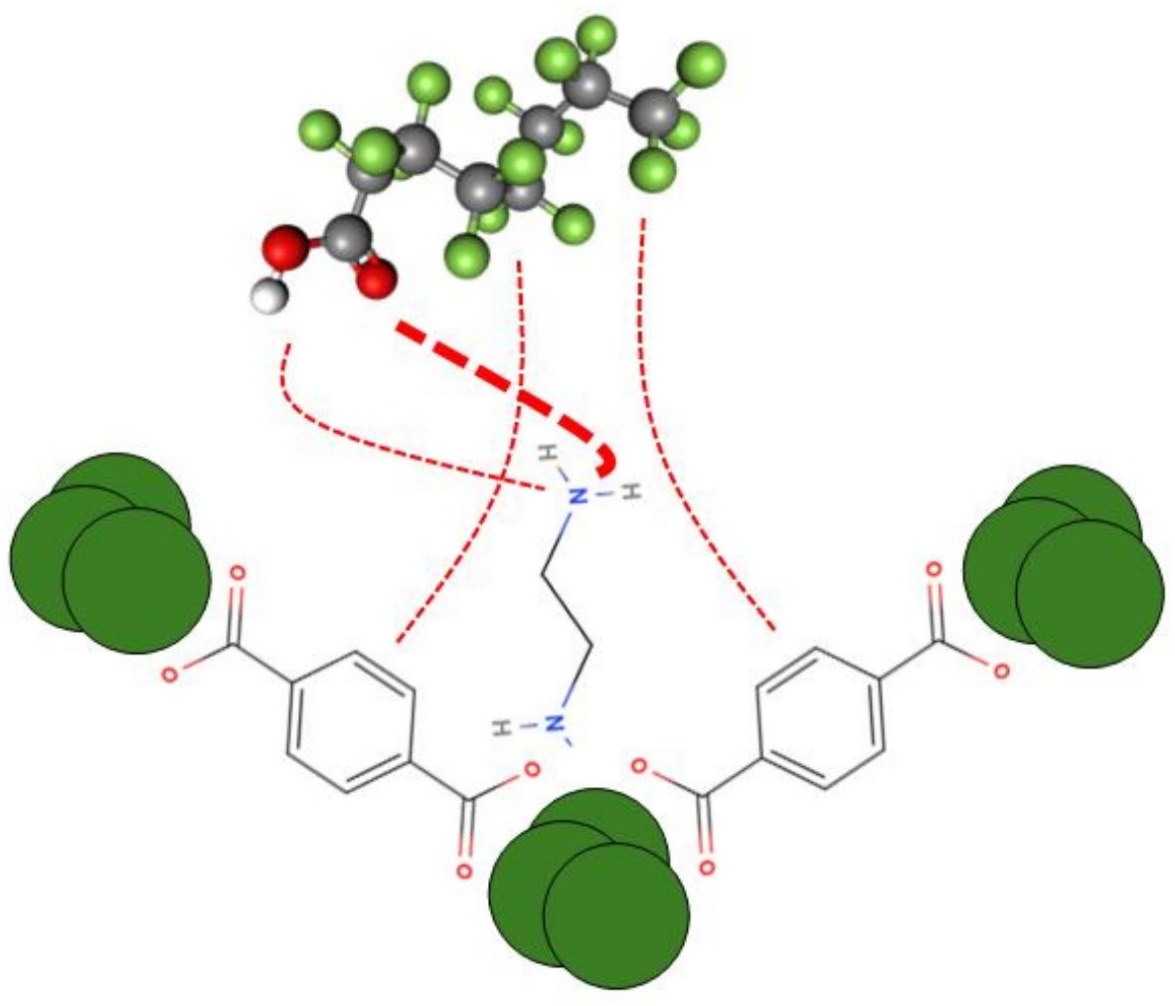
Schematic illustration of the PFOA adsorption mechanism onto NW-101 adsorbent.

This phenomenon can be explained based on the zero charge (PZC) point. The PZC was measured to be 6.5 and 6.3 for the C-101 and W-101, and 5.2 for the NW-101^55^. At pH below the PZC, the surfaces of MOFs are positively charged, thus providing an advantage for adsorbing PFOA, which has a large electron cloud at its head. The structural characterization techniques further affirmed the effect of pH on adsorption. The absence of significant differences was observed in the XRD patterns of the MOFs before and after adsorption; this indicates that the MOF frameworks remain intact and no effect was exerted by scientific structural defects. Substantial changes were observed in the absorption band intensity of the –NH_2_ functional group in the FTIR spectra of NW-101. These changes indicate that amine groups are probably the ones that are the main active sites for adsorption. Under acidic conditions, being protonated, amine groups develop a positive surface onto which PFOA can adsorb, indicating the predominant contribution of electrostatic forces that drive PFOA uptake by NW-101^56^. Besides, the fluorinated tail of PFOA may interact with the aromatic part of the organic linker in MOFs, possibly inducing a π–π interaction. The hydrogen bonding interaction of headgroup acid of PFOA with the amine groups of MOFs may be expected to contribute to the adsorption. Figure 12 shows the schematic illustration of the adsorption mechanism.

### Adsorbents reusability

**Fig. 13:**
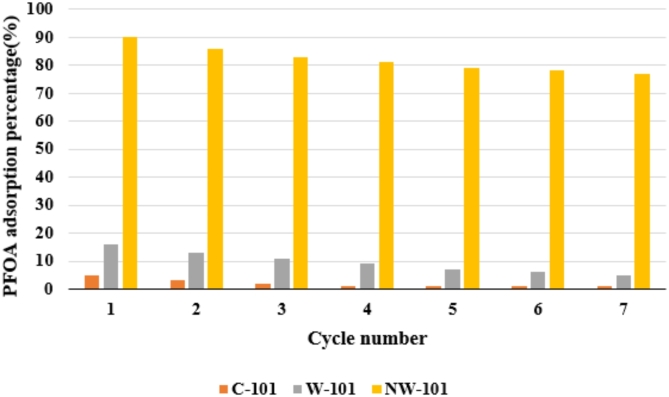
Recovery study of adsorbents for PFOA adsorption process.

Seven adsorption–desorption cycles were conducted at neutral pH 7 for the as-synthesized adsorbents’ reusability efficiency, the outcome of which is presented in Fig. 13. The NW-101 always showed better removal efficiency for PFOA in every cycle, whereas much less difference was seen in W-101 and C-101 removal efficiency over the cycles.

The results showed that NW-101 is more stable and reusable as an absorbent with potential for long work within the environment. The better degree of reusability for NW-101 may be explained by the amine-grafted structure giving it durability and retention of the adsorbing sites under repeated uses in neutral conditions.

### Comparison with previous studies

In the course of our study, we demonstrated that the amine-grafted MIL-101(Cr) (NW-101) exhibited the highest PFOA sorption capacity of 698.4 mg g^−1^, which is significantly greater than other material’s PFOA sorption capacities in the literature. For instance, Kong et al.^51^ reported a PFOA adsorption of 76.59 mg g^−1^ using the derivatization of a F-functionalized MOF containing in-situ-grown TiO_2_. The research conducted by Hu et al.^52^, in their synthesis of DUT-5-2 MOFs through microwave-assisted synthesis reported a PFOA adsorption of 98.2 mg g^−1^. Azmi et al.^54^ used polymer-assisted modified MIL-96(Al) to obtain a PFOA concentration of 200 mg g^−1^. In comparison, the NW-101, produced in the current report, showed far superior PFOA limits as an adsorbent than those materials, showing its potential use as an adsorbent to remove PFOA.

An important breakthrough from this work is the use of waste materials for the synthesis of MOFs. Specifically, the use of PET bottles as a ligand source and waste Cr(VI) as a metal precursor are environmentally friendly alternatives that lower the cost, and pollution footprint and address the issues of plastic and heavy metal waste at the same time. Similarly, Lo et al.^37^ explored the use of waste PET bottles to synthesize nanoporous MOFs such as MIL-47, MIL-53 and MIL-101, but primarily reporting on hydrogen storage, not pollutant adsorption. Ren et al.^38^, also synthesized Cr-based MOFs from PET waste, mainly for hydrogen storage. This study builds on this idea and shows that these MOFs can also be directly used for adsorption of PFOA.

The synthesis of conventional MOFs, as exemplified by MIL-101(Cr), generally requires high purity of chemicals or hazardous reagents like hydrofluoric acid (HF) which creates environmental risks. This study adopts a more sustainable synthesis approach, using nitric acid in lieu of HF, therefore, enhancing environmental safety and scalable production. While Deleu et al.^39^ investigated the use of PET waste resource, they did not focus on the use of industrial chromium waste. The work of this study further expands on that approach, addressing the use of Cr(VI) from industrial waste helps manage the dual agenda of waste utilization and PFOA removal.

General observations of the Langmuir adsorption model and pseudo-second-order kinetics align with the reported underlying mechanisms for MOF-based adsorbents in PFOA removal from other studies, establishing a more robust shared understanding of the well-understood phenomena. Upon these observations NW-101 reached equilibrium quickly (~ 7 min), that would match other well-regarded high-performance MOFs that observe high kinetics^51,52^.

## Conclusions

To put it in a nutshell, despite the existence of several techniques to eradicate persistent organic contaminants such as perfluorooctanoic acid (PFOA) from aqueous media, the threat posed by these compounds to the environment and human health is so severe that there is an urgent need to provide effective solutions to the aforementioned issue. Here, the authors highlighted the potential of three different adsorbents, MIL-101(Cr) fabricated from pure chemicals (C-101), MIL-101(Cr) synthesized from waste materials (W-101), and amine-grafted MIL-101(Cr) made from waste materials (NW-101), for the removal of PFOA from contaminated water. Using PET bottles as a ligand source, wastewater containing Cr(VI) as the metal source, and nitric acid as the mineral agent to produce MIL-101(Cr) was an innovative strategy that considerably reduced the cost and environmental impact of the technique… The findings showed that the PFOA adsorption capacity of NW-101 was the highest (698.4 mg g^−1^), with the adsorption process completed in less than 7 min, and NW-101 outperformed C-101 and W-101. The specific surface areas of C-101, W-101, and NW-101 were 3341, 2767, and 2374 m^2^ g^−1^, respectively. In addition, the Langmuir isotherm showed that it was the fitting one, and that the PFOA uptake was best represented by the pseudo-second order kinetic model.

While this study showed that MIL-101(Cr) derived from waste can be effective in removing PFOA, certain limitations should also be acknowledged. The scaling of the synthesis process might not be simple because of the varying quality of waste-derived materials. Further real-life stability tests are required to assess the performance of the adsorbent over a registered period. Addressing these limitations in future studies will further strengthen the practical applicability of this approach for large-scale wastewater treatment.

Consequently, the results of this study offer a new, low-cost, and recyclable strategy for MIL-101(Cr) fabrication with earth-abundant and impure waste materials as precursors, and a practical approach to the synthesis and purification of PFOA adsorbents for wastewater treatment, opening the way for the sustainable elimination of PFOA from water sources. Our research will have far-reaching consequences as we attempt to confront global environmental problems, particularly water pollution, using new earth-abundant materials for clean and sustainable technological solutions.

## Data Availability

All data generated or analyzed during this study are included in this published article.
